# A meta-analysis of the effects of nitrogen fertilizer application on maize (*Zea mays* L.) yield in Northwest China

**DOI:** 10.3389/fpls.2024.1485237

**Published:** 2025-01-07

**Authors:** Yuanbo Jiang, Haiyan Li, Wenqiong Ma, Wenjing Yu, Junxian Chen, Yalin Gao, Guangping Qi, Minhua Yin, Yanxia Kang, Yanlin Ma, Jinghai Wang, Liting Xu

**Affiliations:** College of Water Conservancy and Hydropower Engineering, Gansu Agricultural University, Lanzhou, China

**Keywords:** nitrogen fertilizer, maize yield, meta-analysis, Northwest China, influencing factor

## Abstract

Nitrogen fertilizer application is an important method for the production of high-quality maize. However, nitrogen fertilizer addition patterns vary according to regional climate, field management practices, and soil conditions. In this study, a meta-analysis was used to quantify the yield effects of nitrogen addition on maize, and meta-regression analysis and a random forest model were used to study the main factors affecting the yield effects of nitrogen addition on maize. The results showed that nitrogen addition significantly increased maize yield by 50.26%–55.72%, and a fluctuating increasing trend was observed with the advancement of the experimental year. The increase in maize yield upon nitrogen addition was the highest in Gansu Province, and showed a decreasing trend with the rise in average annual temperature, but did not change significantly with the average annual precipitation. Among the field management factors, the increase in maize yield was better with the variety Qiangsheng 51, topdressing at the jointing and tasseling stages (JS, TS), nitrogen application rate of 175–225 kg·ha^-1^, and controlled release of nitrogen fertilizer and urea (CRNFU) or the application of a combination of organic and inorganic nitrogen (OIF). Moreover, the positive effects of nitrogen fertilizer application on maize yield improved with soil pH, organic matter, available potassium, available phosphorus, and total nitrogen content; decreased with soil carbon and nitrogen ratio and available nitrogen (AN) content; and were enhanced in chestnut soil, clay, and at a bulk density of 1.2–1.4 g·cm^-3^. Random forest model and multifactorial optimization revealed that the effects of nitrogen addition on maize yield in Northwest China were primarily influenced by experimental year, variety, soil type, AN, and soil pH.

## 1 Introduction

Maize (Zea mays L.) is a rich source of various nutrients such as starch, protein, and fat. Mainly grown in countries such as the United States, China, Brazil, and Argentina, it is an economically important food, feed, and industrial raw-material crop worldwide (Yan et al., 2015; Li et al., 2020). China ranks second in global maize production, with an annual output of 260 million tonnes. However, in recent years, maize imports have surged due to rising domestic demand and low production. In 2021, maize imports in China reached 28.4 million tonnes, which accounted for approximately 10% of the total demand (Luo et al., 2023). Northwest China is the core region in the Belt and Road Strategy of China, the Western Development, and the high-quality development of the Yellow River Basin (Wu et al., 2023), covering 33.1% China’s total land area. With 10% of the national water resources, the western region is responsible for 12% of China’s food production, which plays an important role in guaranteeing national food security (Shan et al., 2021). Nonetheless, because of the limited possibility for expansion of maize acreage, China must increase its maize production (output per unit area) if it needs to achieve self-sufficiency. Fertilizer application is an effective measure to achieve an increase in maize yields, and in Northwest China, high fertilizer inputs have provided a significant yield advantage to growing maize (Wu et al., 2022), with maize yields reaching 8.9 t·ha^-1^ in 2018, far exceeding the national average of 6.1 t·ha^-1^ (Qiao et al., 2022). However, the large input of fertilizers not only puts the environment constantly under pressure but also contradicts the national strategic plan to discontinue the use of chemical fertilizers and pesticides by 2030 (Sainju et al., 2003; Wang et al., 2016a, 2022). Therefore, promoting the use of highly efficient fertilizers and realizing the organic combination of production and ecology in maize production is of great significance for sustainable agriculture development in Northwest China.

Maize growth and development are influenced by both environmental and genetic factors (Jiang et al., 2022). Among various environmental factors, fertilizers play a crucial role in enhancing maize yield, wherein nitrogen, as an essential mineral nutrient and the basis of vital substances, is directly involved in the synthesis of a wide range of plant organic matter (Li, 2015). Nitrogen fertilizer application is the most direct and effective way to increase the content of available nitrogen in the soils of maize-growing areas. However, studies suggest that although nitrogen fertilizers are the most produced and used fertilizers in China, their utilization efficiency is approximately 30% (Li, 2015). Excessive application of nitrogen fertilizers causes soil acidification, nitrogen redundancy, leaching, and nitrogen deposition while significantly reducing maize yield and nitrogen-use efficiency (Cassman et al., 2002; Lv et al., 2007; Guo et al., 2010; Liu et al., 2023). Furthermore, variations in factors such as regional climate, field management practices, and soil conditions influence how nitrogen addition affects maize yield. The effects of nitrogen addition on maize yield vary in different ecological zones in Jilin Province, China, with the highest yield increase notes in the eastern region, followed by the central region, and a significantly lower yield increase in the western region (Wang et al., 2016c). In India and northern Florida, USA, optimum maize yields of 4.5–4.6 t·ha^-1^ and 12.2 t·ha^-1^ were achieved at recommended nitrogen application rates (NARs) of 150 kg·ha^-1^ and 264 kg·ha^-1^, respectively (Zamora-Re et al., 2020; Kumar et al., 2021). Dirks and Bolton (1981) showed that more than 80% of maize yield could be explained by the temperature of Brookston clay soils in Canada. Frequent low-intensity precipitation and high-temperature environments can promote crop response to nitrogen fertilizers by reducing leaching and volatile losses and enhancing adsorption (Xie et al., 2013). Bacon et al. (2016) found that in Timor-Leste, high temperatures and no nitrogen fertilizer application increased maize yields in colder, high-altitude areas and reduced yields in warmer coastal areas. They also noted that increasing temperatures after nitrogen application resulted in a decreasing trend in maize yields in all regions.

Among the field management factors, the frequency and period of nitrogen application exhibited significant effects on maize yield. Adriaanse and Human (1993) showed that maize yield was lower when nitrogen fertilizer was applied two or three times compared to a single application under dry cropping conditions. Additionally, maize yield was not altered upon delaying nitrogen application to the 10th (V10) or 11th leaf nutritive growth period, but it showed a decreasing trend upon delaying nitrogen application to the tasseling or silking stage (Scharf et al., 2002; Da Silva et al., 2005; Walsh et al., 2012). However, Lv et al. (2012) found that nitrogen application at the jointing stage, V10, and 10 days after anthesis increased maize yield. In addition, the location of nitrogen application and type of nitrogen fertilizer largely influenced maize yield. Sepahvand et al. (2014) found that strip- and spread-applied nitrogen fertilizers increased maize kernel yield by 91.4% and 3.9%, respectively, compared to no nitrogen fertilizer application. The combination of organic and inorganic nitrogen can significantly improve soil nitrogen supply capacity, promote biological nitrogen fixation, reduce nitrogen loss, and increase nitrogen-use efficiency (Han et al., 2014). Chinese farmers typically apply around 209 kg·ha^-1^ of nitrogen fertilizers to maize fields (Wang, 2007), which exceeds the recommended 150–180 kg·ha^-1^ (Li, 1998). The increase in nitrogen fertilizer application does not mean a simultaneous increase in yield and nitrogen-use efficiency; when nitrogen fertilizer application was < 60 kg·ha^-1^, the maize yield was 6.21 t·ha^-1^ and the nitrogen fertilizer-utilization rate was 40.2%; and when nitrogen fertilizer application was > 240 kg·ha^-1^, maize yield dropped to 5.52 t·ha^-1^ and the nitrogen fertilizer-utilization rate dropped sharply to 14.4% (Zhang et al., 2008). However, Wang et al. (2018) found that maize yield and nitrogen-recycling efficiency under mulch drip irrigation in Inner Mongolia, China, first showed an increasing and then a decreasing trend with the increase in nitrogen fertilizer application, and the optimal range of nitrogen application to ensure high maize yield was 240–253 kg·ha^-1^. In addition to the report on how to ensure rational field management, in terms of soil environmental factors, few scholars have found that coarse-grained soils usually ensure higher yields than fine-grained soils in humid environments, while clay soils (high water-holding properties) are more capable of providing higher crop yields under arid conditions (Tremblay et al., 2012). A single application of chemical fertilizers can also increase maize yield by increasing the content of soil organic matter (OM), but when nitrogen application is > 360 kg·ha^-1^, the content of soil OM increases slowly, which affects the increase in maize yield (Liu et al., 2021). Studies showed that maize yield was negatively correlated with soil bulk density and pH, and positively correlated with soil quick-acting nutrients, such as alkaline dissolved nitrogen and available phosphorus (Cheng et al., 2024). Overall, the yield characteristics of maize fertilized using nitrogen fertilizers are affected by the complex effects of regional climate, field management practices, and soil conditions.

While several studies have examined the impact of nitrogen addition on maize yield, most were conducted at fixed locations, complicating the interpretation of these effects and their determinants over broader regions. Additionally, there are fewer studies on the effects of integrating the regional climate, field management practices, and soil environmental factors on the yield of maize fertilized using nitrogen fertilizers. In addition, the following questions remain unanswered: Are there specific environmental conditions that ensure the positive effects of nitrogen addition on maize yield, and if so, do they influence the strength of the positive effects? What are the most important environmental factors affecting nitrogen-fertilized maize field management? In this regard, meta-analysis—a statistical method for comparing and integrating the results of multiple studies—can reveal the potential factors affecting the target on a regional or global scale through comprehensive quantitative analysis of existing experimental data (Wang et al., 2019; Zhang et al., 2022b). The results of a meta-analysis can be supplemented by meta-regression analysis, which elucidates the main factors affecting the dependent variable and the magnitude and direction of the effect (Benitez-Lopez et al., 2017), and the random forest model, which is an efficient combinatorial classification method to rank the importance of factors affecting the dependent variable (Terrer et al., 2021). Thus, this study explored the effects of nitrogen addition on maize yield in Northwest China using a meta-analysis based on published data, and screened the main factors affecting the changes in maize yield upon nitrogen addition using meta-regression analysis and the random forest model, with the aim of (1) quantifying the effects of nitrogen addition on maize yield in Northwest China; (2) analyzing how this effect varies with environmental conditions and farmland management practices; and (3) providing a reference for further improvement of maize yield and sustainable agriculture development in nitrogen application environments.

## 2 Materials and methods

### 2.1 Data sources and collection criteria

**Figure 1:**
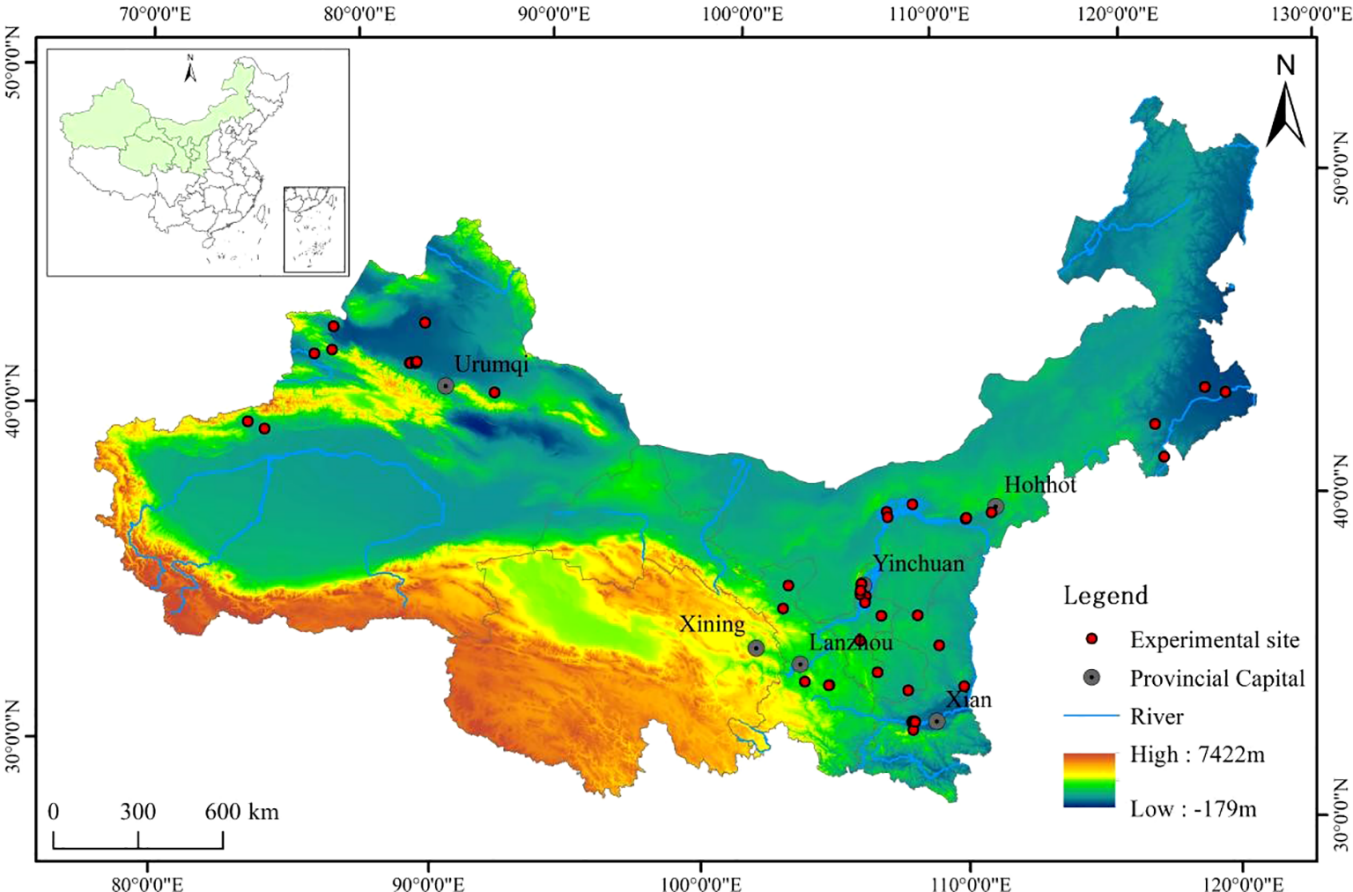
Location distribution of cases in this meta-analysis.

**Figure 2:**
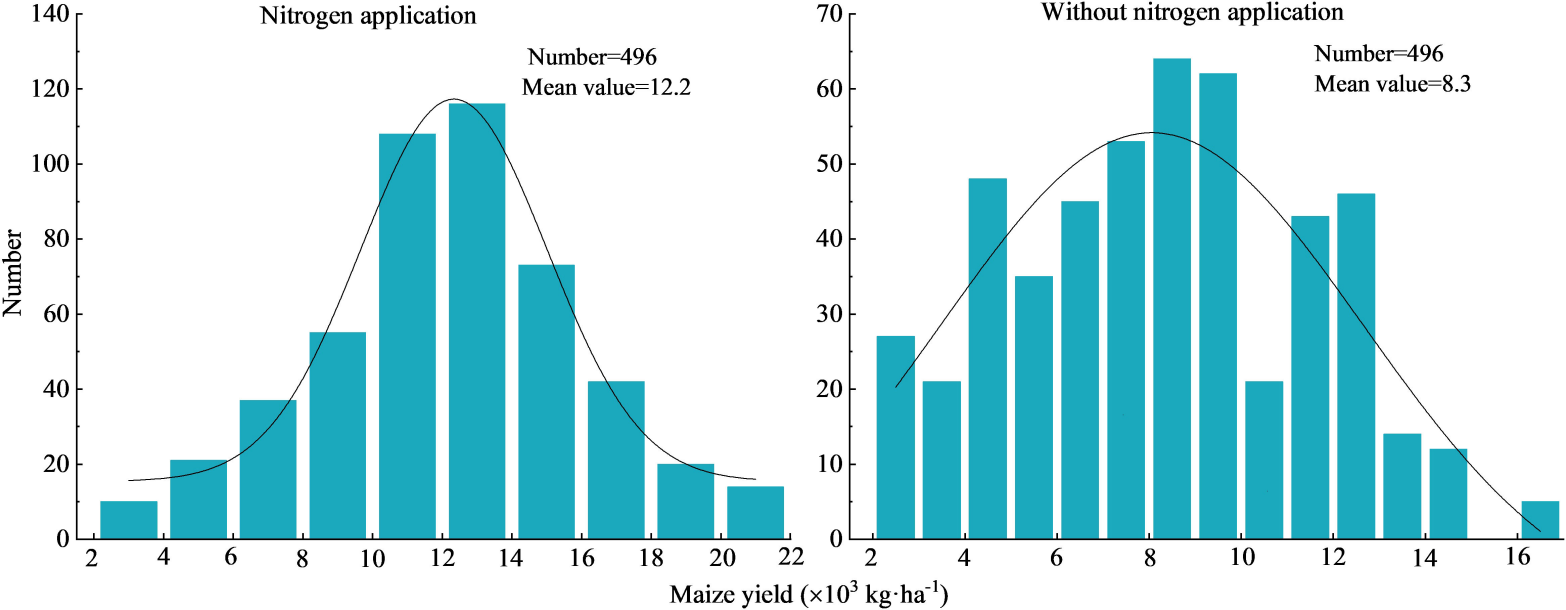
Frequency distribution of maize yield with and without added nitrogen conditions.

In this study, we identified peer-reviewed studies focusing on the effects of nitrogen fertilizer application on maize yield in the China National Knowledge Infrastructure (CNKI, http://www.cnki.net/) and the Web of Science (https://www.webofscience.com/) in December 2022, using the keywords corn/maize, nitrogen application, yield, Northwest China, Gansu, Inner Mongolia, Ningxia, Qinghai, Xinjiang, and Shaanxi. To minimize data impact, papers were strictly screened based on the following criteria: (1) the trial must be a field trial and the trial site is located in Northwest China (**Figure 1**); (2) the trial must have a nitrogen application treatment and no nitrogen application treatment, with the same experimental conditions except for the nitrogen application factor; (3) the yield data of the two treatments are explicitly mentioned and their averages and standard deviations are given or can be obtained by computation; and (4) the type of nitrogen application, amount, period, and data on temperature, precipitation, irrigation, test year, soil, etc. can be obtained for one or more of these. Based on the above criteria, a total of 53 papers (containing 496 pairs of yield data) were obtained, and the data were derived from tables or figures in these papers (obtained using Web Plot Digitizer software).

### 2.2 Data classification and processing

#### 2.2.1 Data classification

As the effects of nitrogen fertilizer application on maize yield are directly or indirectly affected by the external environment and genetic characteristics, a subgroup analysis of each factor was used to examine its effect on maize yield. The classification criteria for each factor were as follows: (1) five provinces of Northwest China [Gansu, Inner Mongolia, Ningxia, Xinjiang, and Shaanxi (no data were retrieved for Qinghai)]; (2) nitrogen fertilizer types [controlled release of nitrogen fertilizer (CRNF), controlled release of nitrogen fertilizer and urea (CRNFU), urea, urea and ammonium nitrate (UAN), organic and inorganic nitrogen fertilizer (OIF), and fertilizer for maize drip irrigation (FMDI)]; (3) NAR (< 125, 125–175, 175–225, 225–275, 275–325, 325–375, 375–425, and ≥ 425 kg·ha^-1^); (4) topdressing (yes or no); (5) application stage [jointing stage (JS), tasseling stage (TS), grain filling stage (GS), JS + TS, JS + GS, and JS + TS + GS]; (6) average annual precipitation (AAP; < 200, 200–400, and ≥ 400 mm); (7) average annual temperature (AAT; < 4, 4–8, 8–12, and ≥ 12 °C); (8) cropping systems (maize succession cropping and winter wheat–summer maize rotation); (9) planting density (< 5×10^4^, 5–8×10^4^, 8–11×10^4^, and ≥ 11×10^4^ plants·ha^-1^); (10) maize varieties with the largest sample sizes (Xianyu 335, Zhengdan 958, LuoDan 9, Qiangsheng 51, ShanDan 609, and Xianfeng 335); (11) soil types according to the China Soil Classification and Codes 2009 (GB/T 17296-2009, 2009) [meadow soil (MeS), moisture soil (MS), irrigated desert soil (IDS), anthropogenic-alluvial soil (AS), cinnamon soil (CiS), heilu soil (HS), loessal soil (LS), sierozem (Si), desert gray soil (DGS), chestnut soil (CS), and new sediment soil (NSS)]; (12) soil texture [clay (C), loam (L), sandy soil (SS), sandy loam soil (SLS), and silty clay loam (SCL)]; (13) soil bulk density (BD; < 1.2, 1.2–1.4, 1.4–1.6, and ≥ 1.6 g·cm^-3^); (14) soil pH (< 7.5, 7.5–8.5, and ≥ 8.5); (15) soil OM (< 10, 10–15, 15–20, and ≥ 20 g·kg^-1^); (16) soil carbon–nitrogen ratio (C/N; < 9, 9–11, and ≥ 11); (17) soil total nitrogen (TN; < 0.6, 0.6–1.0, and ≥ 1.0 g·kg^-1^); (18) soil available nitrogen (AN; < 20, 20–35, and ≥ 35 mg·kg^-1^); (19) soil available phosphorus (AP; < 15, 15–30, 15–30, and ≥ 30 mg·kg^-1^); (20) soil available potassium (AK; < 100, 100–170, and ≥ 170 mg·kg^-1^); (21) irrigation (yes or no); and (22) irrigation method [drip irrigation (DI), flood irrigation (FI), and border irrigation (BI)].

#### 2.2.2 Data Processing

1. Partial factor productivity of nitrogen (*PFP_N_*, kg·kg^-1^) was calculated using the following formula:


(1) $$PFP_{N}=Y/N$$

where *Y* is the yield (kg·ha^-1^), and *N* is the NAR (kg·ha^-1^).

2. Soil C/N was determined as follows:


(2) SOC=0.58×SOM

(3) C/N=SOC/TN

where *SOC* is the soil organic carbon content (g·kg^-1^), *SOM* is the soil organic matter content (g·kg^-1^), 0.58 is the conversion coefficient, and *TN* is the soil total nitrogen content (g·kg^-1^).

### 2.3 Meta-analysis

#### 2.3.1 Calculation of effect size and weight for individual cases

In this study, logarithmic response ratio (ln*R*) was used to characterize the effects of nitrogen addition on maize yield, with the advantage that data dimensions can be eliminated, and the range can extended from negative infinity to positive infinity (Hedges and Gurevitch, 1999):


(4) $$\ln R=\ln(X_{a}/X_{c})$$

where *R* is the response ratio, *X_a_* is the average yield of maize upon nitrogen addition (kg·ha^-1^), and *X_c_* is the average yield of maize without nitrogen addition (kg·ha^-1^).

The variance within the case (*v_i_*) was calculated as follows:


(5) $$v_{i}=\frac{S_{a}^{2}}{N_{a}X_{a}^{2}}+\frac{S_{c}^{2}}{N_{c}X_{c}^{2}}$$

where *v_i_* is the within-case variance for a single case; *S_a_* and *S_c_* are the standard deviations of maize yield with and without nitrogen application, respectively; and when the standard deviation was missing in the study, it was calculated using the methodology described by Gattinger et al. (2012). *N_a_* and *N_c_* are the numbers of replicated trials for maize yield with and without nitrogen application, respectively.

The weight of the *i*th study (*w_i_*) was calculated as follows:


(6) $$w_{i}=1/(v_{i}+\tau^{2})$$

where *τ* ^2^ is the between-case variance.

#### 2.3.2 Calculation of cumulative effect size

In this study, external factors, such as regional climate, field management practices, and soil properties, affected the results, which were considered heterogeneous. Hence, the random-effects model was used to calculate the cumulative effect size ( $\bar{y}$ ).


(7) $$\bar{y}=\sum_{i=1}^{k}w_{i}y_{i}/\sum_{i=1}^{k}w_{i}$$

(8) $$SE=\sqrt{\frac{1}{\sum_{i=1}^{k}w_{i}}}$$

(9) $$95\%CI=\bar{y}\pm 1.96SE$$

where 95% *CI* is the 95% confidence interval, and *SE* is the overall standard error.

To directly represent maize yield increase upon nitrogen application in response to each factor, ln*R* was converted to the rate of yield improvement (*Z*) over the control treatment (Hedges and Gurevitch, 1999).


(10) Z=[exp(lnR)−1]×100

#### 2.3.3 Analysis of influencing factors

If the heterogeneity test found that *Q_t_* did not obey the chi-squared distribution (*P* < 0.05), we introduced explanatory variables, subgroup analysis, or meta-regression analysis (Buhmann et al., 2015; R Core Team, 2018). Additionally, significant heterogeneity (*Q_M_*) caused by a variable (*P* < 0.05) indicated that the factor exhibited significant effects on maize yield upon nitrogen application.

#### 2.3.4 Model test

In this study, publication favoritism of the model was tested using the coefficient of insecurity. If the coefficient of insecurity was > 5k+10, the results of the study were not affected by publication favoritism and were highly credible (Xiang et al., 2017).

### 2.4 Data analyses

The “metafor” package of R software (4.3.0) was used for meta-analysis, the “randomForest” package for random forest analysis, the “glmulti” package for multivariate optimization, Microsoft Excel 2016 for data preparation, and Origin 2021 for plotting.

## 3 Results

### 3.1 Data distribution and overview

**Figure 3:**
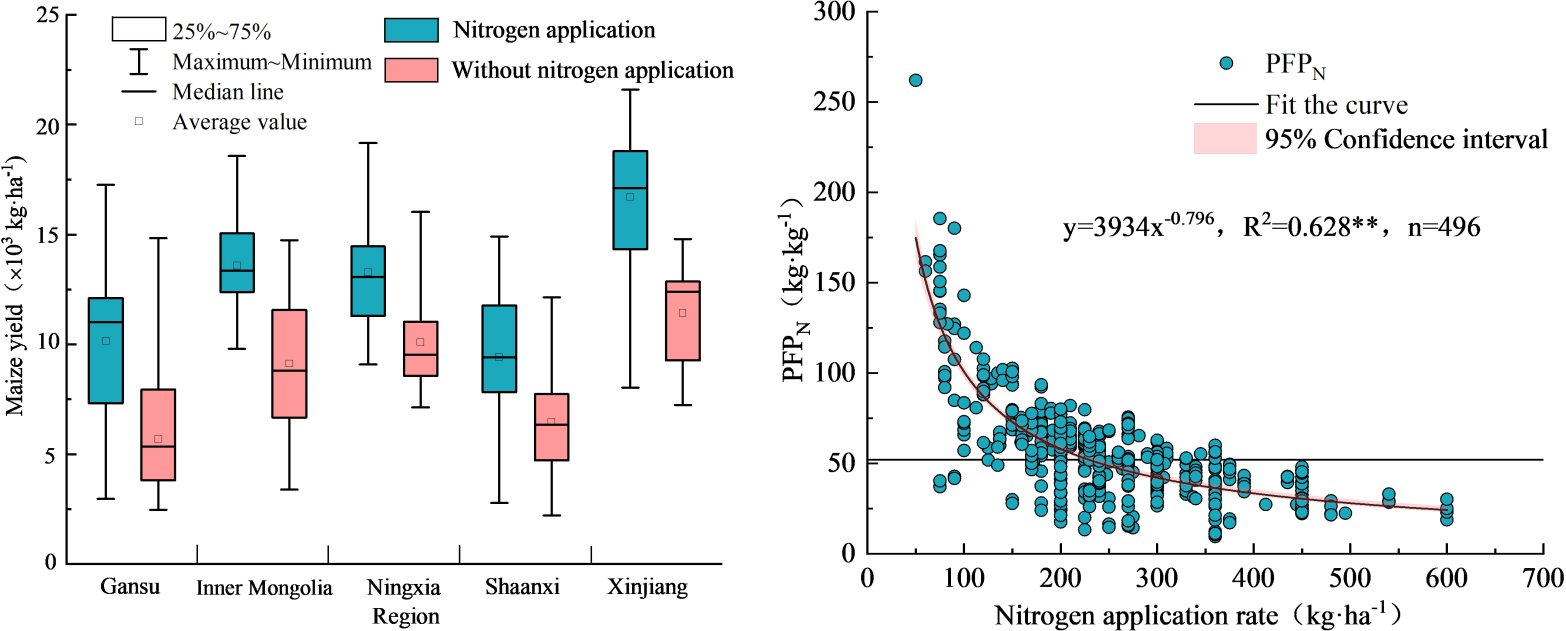
Regional distribution of maize yield and the relationship between nitrogen application and PFP_N_. ** in the figure represents significant correlation at the *P*<0.01 level.

Influenced by factors, such as regional climate, field management practices, and soil conditions, maize yield exhibited large variability with and without nitrogen addition and showed normal distribution (**Figure 2**). Maize yield upon nitrogen addition ranged from 2.8×10^3^ kg·ha^-1^ to 21.6×10^3^ (average: 12.2×10^3^ kg·ha^-1^), and that without nitrogen addition ranged from 2.2×10^3^ kg·ha^-1^ to 16.0×10^3^ kg·ha^-1^ (average: 8.3×10^3^ kg·ha^-1^). As shown in **Figure 3**, the median maize yields in the five provinces with and without nitrogen addition were 10.2×10^3^–17.2×10^3^ kg·ha^-1^ and 5.3×10^3^–12.4×10^3^ kg·ha^-1^, respectively. This study on the relationship between NAR and partial factor productivity of nitrogen found that the two were negatively correlated in a power function type, with a fitting curve of y = 3934x^-0.796^ and *R* ^2^ of 0.628, indicating that maize yield per unit of nitrogen application gradually decreased with the amount of nitrogen applied.

### 3.2 Bias testing

**Figure 4:**
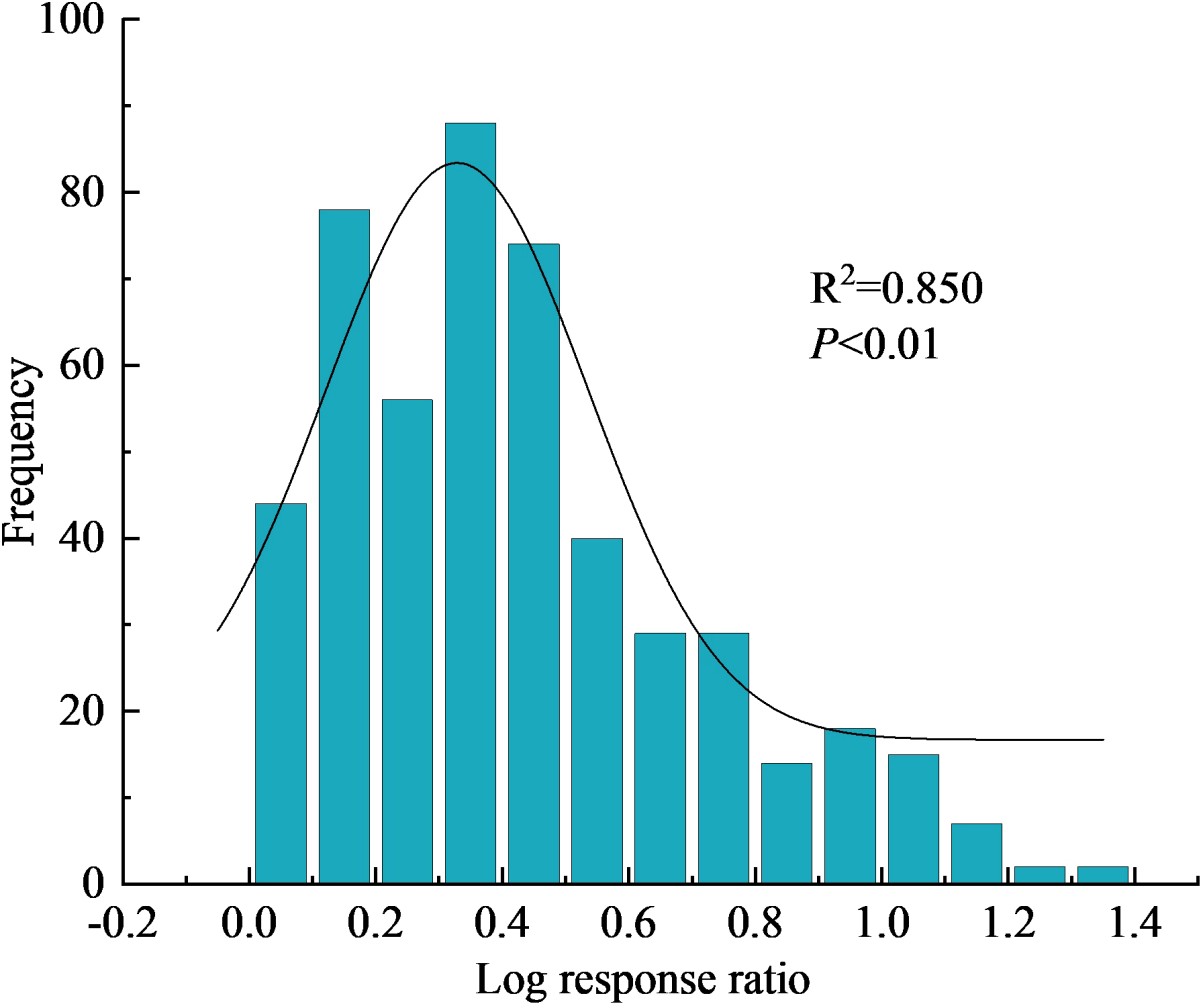
Frequency distribution of maize yield effect size in response to nitrogen application.

In this study, the collected yield data pairs were tested for bias by fitting the yield effect value distribution to a Gaussian function (**Figure 4**). The Kolmogorov-Smirnov test showed that the frequency distribution of the yield effect value under different fertilization conditions did not obey normal distribution (*P* < 0.01). Therefore, lnR and 95% CI generated by the non-parametric estimation method (bootstrap resampling; 1000 times) were used for data analyses (Wang et al., 2019).

### 3.3 Cumulative effect size of nitrogen application on yield response of maize and test for publication preference

**Table 1**, **Figure 5** demonstrate that the cumulative effect size of nitrogen addition on maize yield was 0.4255 (95% CI, 0.4072–0.4429), that is, compared to no nitrogen addition, under 95% CI, nitrogen addition increased maize yield by 50.26%–55.72% (average: 53.04%). In addition, the variance between cases accounted for 99.6% of the total variance of the model I^2^, and the random-effects model indicated highly heterogeneous data (*Q_t_* = 44580.2262, *P_Q_* < 0.0001). Therefore, it was necessary to introduce explanatory variables to study the factors affecting maize yield on nitrogen. The failure safety factor of 5657075 was much larger than that of 5k + 10 (n is the sample size), indicating that the test results were not affected by publication bias, and the results exhibited high credibility.

**Table 1:** Cumulative effect size analysis of nitrogen application on maize yield.

$\bar{y}$	k	*P* value	95% LCI	95% UCI	I^2^(%)	heterogeneity test		K	5k+10
						Q_t_	P_Q_		
0.4255	496	<0.0001	0.4072	0.4429	99.69	44580.2262	<0.0001	5657075	2490

k is the sample size, 95% LCI is lower limit of 95% confidence interval, 95% UCI is upper 95% confidence interval. I^2^ represents the between-case variance as a percentage of the total model variance. K is the coefficient of insecurity.

**Figure 5:**
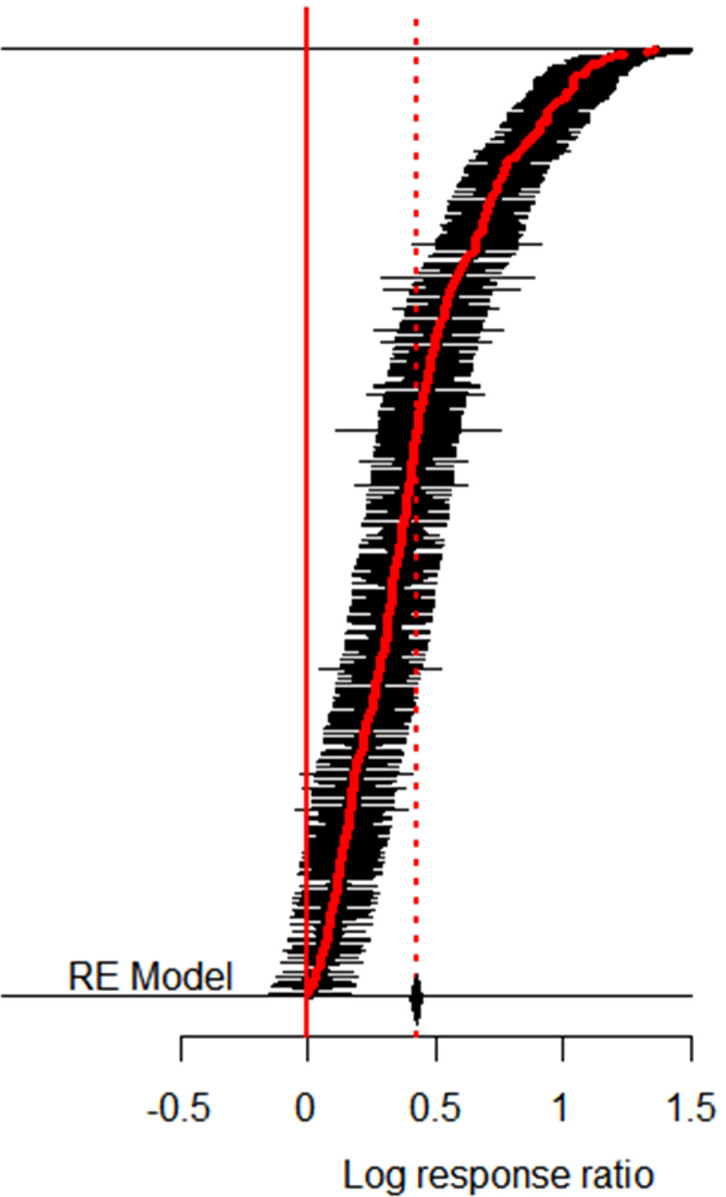
Distribution of individual effect sizes and their cumulative effect size of nitrogen application on maize yield response cases. The black diamonds in the figure represent the cumulative effect size of nitrogen application on the yield response of maize, and the black shading represents the distribution of effect size for individual cases.

### 3.4 Analysis of the influence factors of nitrogen addition on maize yield change

#### 3.4.1 Regional climate factors

**Figure 6:**
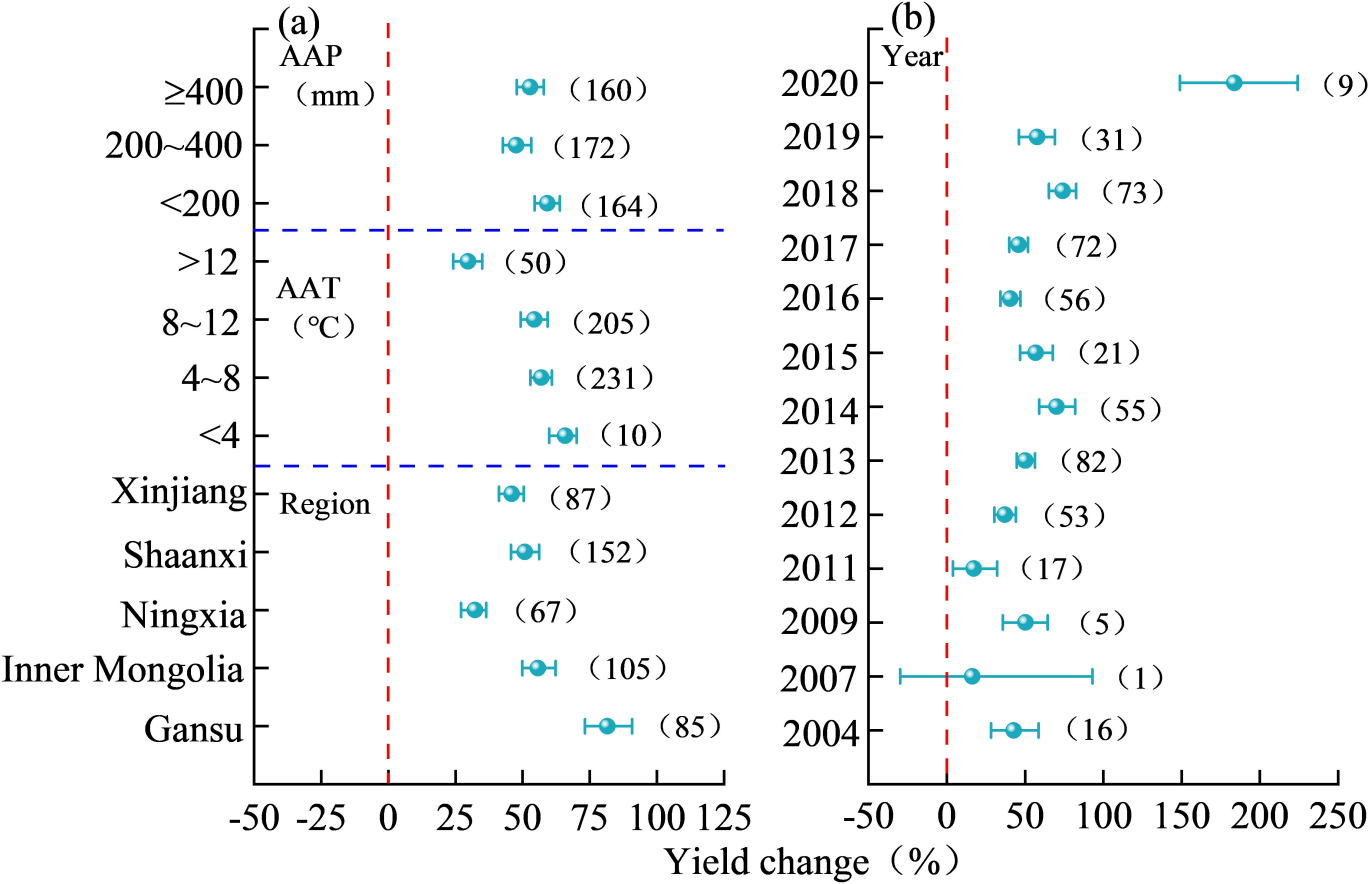
Influence of regional climatic factors on yield changes in nitrogen-applied maize. In **(A)** AAP stands for average annual precipitation, AAT stands for average annual temperature and Region stands for different provinces in China. In **(B)** Year stands for the year of the experiment.

As can be seen from **Figure 6**, regional climate factors significantly influenced the yield effects of nitrogen addition on maize. The difference between the yield-increasing effects of AAP was not significant, with a maximum maize yield increase of 59.27% (95% CI, 54.47%–63.89%) at AAP < 200 mm, and the yield-increasing effect of AAP of ≥ 400 mm being the second highest. The yield-increasing effects of AAT increased with rising temperatures, but the yield-increasing effects of nitrogen application with AAT < 12°C were not significant, and the minimum increase in maize yield was noted at AAT > 12°C, which was 29.64% (95% CI, 24.16%–34.97%). The effects of nitrogen application on maize yield increase in Gansu was 81.59% (95% CI, 73.24%–90.77%), followed by Inner Mongolia, Shaanxi, and Xinjiang, and the lowest in Ningxia. The increase in maize yield upon nitrogen application showed a fluctuating, increasing trend as the year progressed, and the maize yield increase in 2020 was 183.60% (95% CI, 148.93%–224.14%). Overall, the years closer to the present, lower AAP and AAT were conducive to increased maize yield upon nitrogen application.

#### 3.4.2 Field management factors

**Figure 7:**
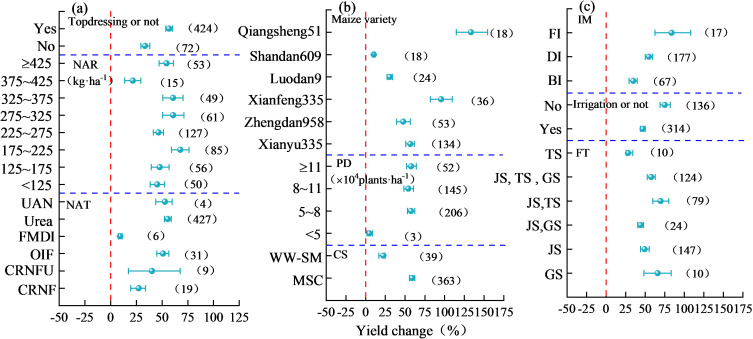
Effect of field management on yield changes in nitrogen-applied maize. In **(A)** NAR stands for nitrogen application rate, NAT stands for nitrogen application type, UAN stands for urea and ammonium nitrate, FMDI stands for fertilizer for maize drip irrigation, OIF for organic and inorganic fertilization, CRNFU for controlled release nitrogen fertilizer and urea, CRNF for controlled release nitrogen fertilizer. In **(B)** PD stands for planting density, CS stands for cropping system, WW-SM stands for winter wheat-summer maize, and MSC stands for maize succession cropping. In **(C)** IM stands for irrigation method, FT for fertilization time, FI for flood irrigation, DI for drip irrigation, BI for border irrigation, TS for tasseling stage, JS for jointing stage, GS for grain filling stage.

The *Q_m_* value (*P* < 0.05) of each field management factor (**Table 2**) suggested that the field management factors significantly affected the yield effect of nitrogen addition on maize. Among the field management factors (**Figure 7**), topdressing significantly increased the yield-increasing effect of nitrogen application by 56.74% (95% CI, 53.56%–60.06%) compared to basal nitrogen application. Moreover, with an increase in NAR, the yield-increasing effects first increased and then decreased gradually. When NAR was 175–225 kg·ha^-1^, the yield-increasing effect was the highest (67.74%; 95% CI, 59.27%–76.17%). However, when the NAR was 375–425 kg·ha^-1^, the yield-increasing effect of nitrogen application began to decrease significantly. Concerning nitrogen application, the yield-increasing effects of urea, UAN, OIF, and CRNFU, followed by CRNF, on maize were high, but not significantly different. Nonetheless, the yield-increasing effect of DI was minimal, and the order for fertilization period was JS + TS > GS > JS + TS + GS > JS > JS + GS > TS, among which the yield-increasing effect at JS + TS was up to 69.67% (95% CI, 59.79%–80.16%). Furthermore, the variety with the highest yield-increasing effect [95.52% (95% CI, 82.08%–109.78%)] was Qiangsheng 51, followed by Xianfeng 335. When the planting density was more than 5×10^4^ plants·ha^-1^, the yield-increasing effects of nitrogen application was significantly high, but later, the effects of increase in planting density was not significant. Compared to winter wheat–summer maize rotation, maize succession cropping exhibited a higher yield-increasing effect [58.84% (95% CI, 55.46%–62.34%). The yield-increasing effect of nitrogen application without irrigation was higher than that with irrigation, and it followed this order: FI > DI > BI.

**Table 2:** Heterogeneity analysis of each influencing factor.

Factor	Q_M_	*P* value	Factor	Q_M_	*P* value
NAT	20.47	0.0010	Maize variety	317.81	<0.0001
NAR	29.48	0.0001	STy	75.35	<0.0002
Topdressing or not	21.51	<0.0001	STe	95.61	<0.0003
FT	17.81	0.0032	Irrigstion or not	40.52	<0.0004
Region	58.75	0.0001	IM	24.47	<0.0005
CS	36.23	0.0001			

NAT represents Nitrogen application type; NAR represents nitrogen application rate; FT represents fertilizing time; CS represents cropping system; STy represents soil type; STe represents soil texture; IM represents irrigation method.

#### 3.4.3 Soil environment

**Figure 8:**
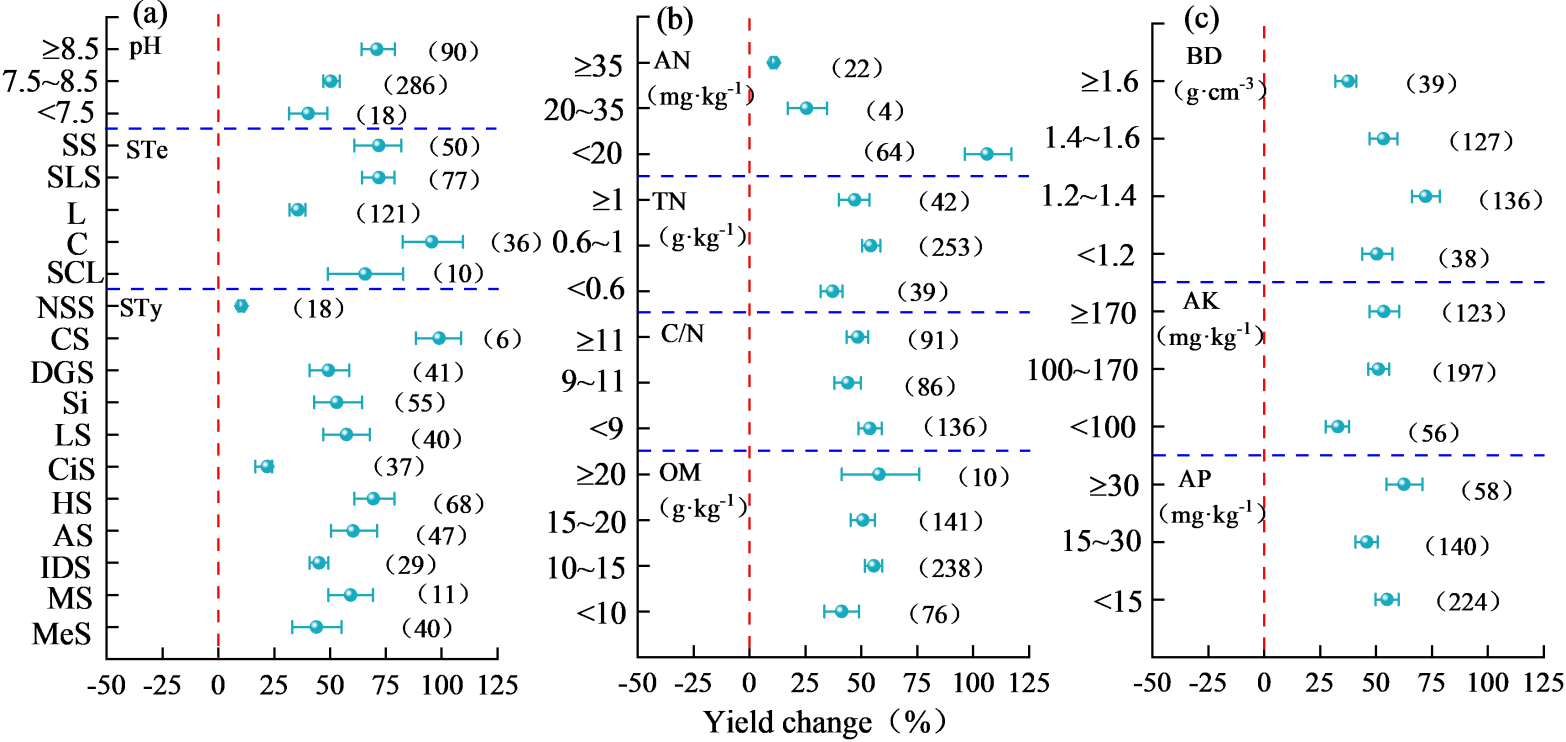
Influence of soil environment on yield changes in nitrogen-applied maize. In **(A)**, STe stands for soil texture, STy stands for soil type, SS stands for sandy soil, SLS stands for sandy loam soil, L stands for loam, C stands for clay, SCL stands for silty clay loam, NSS stands for new sediment soil, CS for chestnut soil, DGS for desert gray soil, Si for sierozem, LS for loessal soil, CiS for cinnamon soil, HS for heilu soil, AS for anthropogenic-alluvial soil, IDS for irrigated desert soil, MS for moisture soil, and MeS for meadow soil. In **(B)** AN stands for available nitrogen, TN for total nitrogen, and OM for organic matter, and in **(C)** BD stands for bulk density, AK stands for available potassium, and AP stands for available phosphorus.

**Figure 8** illustrates that different soil conditions significantly affected the increase in maize yield upon nitrogen addition. With the increase in soil pH, the yield-increasing effects of nitrogen fertilization improved gradually, with the maximum value at pH ≥ 8.5, which was 70.93% (95% CI, 64.03%–79.00%). From the perspective of soil texture, maize showed the highest yield increase of 95.52% (95% CI, 82.45% –109.53%) in C, followed by SL, SS, and SCL, and finally L. In addition, the most significant increase in maize yield upon nitrogen application [98.91% (95% CI, 88.49%–108.57%)] was observed in CS, followed by HS [69.45% (95% CI, 60.87%–78.71%)], and the weakest increase in maize yield was observed in NSS. The yield-increasing effects of nitrogen application on maize gradually decreased with increasing soil AN content, with the highest yield-increasing effect of 106.27% (95% CI, 96.34%–117.19%) at AN content < 20 mg·kg^-1^. A yield-increasing effect of 54.23% (95% CI, 50.35%–58.57%) was observed in maize at 0.6–1 g·kg^-1^ of soil TN. However, with an increase in soil C/N, the yield-increasing effects of nitrogen application decreased gradually, but there was no significant difference in yield increase between soils with different C/N. The yield-increasing effects of nitrogen application increased gradually with increasing soil OM content, with the highest yield-increasing effect of 58.01% (95% CI, 41.30%–75.91%) when the soil OM content was ≥ 20 g·kg^-1^. Furthermore, with the increase in soil BD, the yield-increasing effects of nitrogen application showed an increasing and then a decreasing trend, and the maximum value was recorded at soil BD of 1.2–1.4 g·cm^-3^, which was 72.19% (95% CI, 66.05%–78.57%). With increasing soil AK content, the yield-increasing effects of nitrogen application showed an increasing trend, and the maximum yield-increasing effect [53.50% (95% CI, 47.09%–60.35%)] was noted at ≥ 170 mg·kg^-1^. Moreover, with an increase in soil AP content, the yield-increasing effects of nitrogen application gradually increased, and the maximum yield-increasing effect of maize was 62.53% (95% CI, 54.64%–70.74%) when the soil AP content was ≥ 30 mg·kg^-1^.

#### 3.4.4 Meta-regression analysis

**Figure 9:**
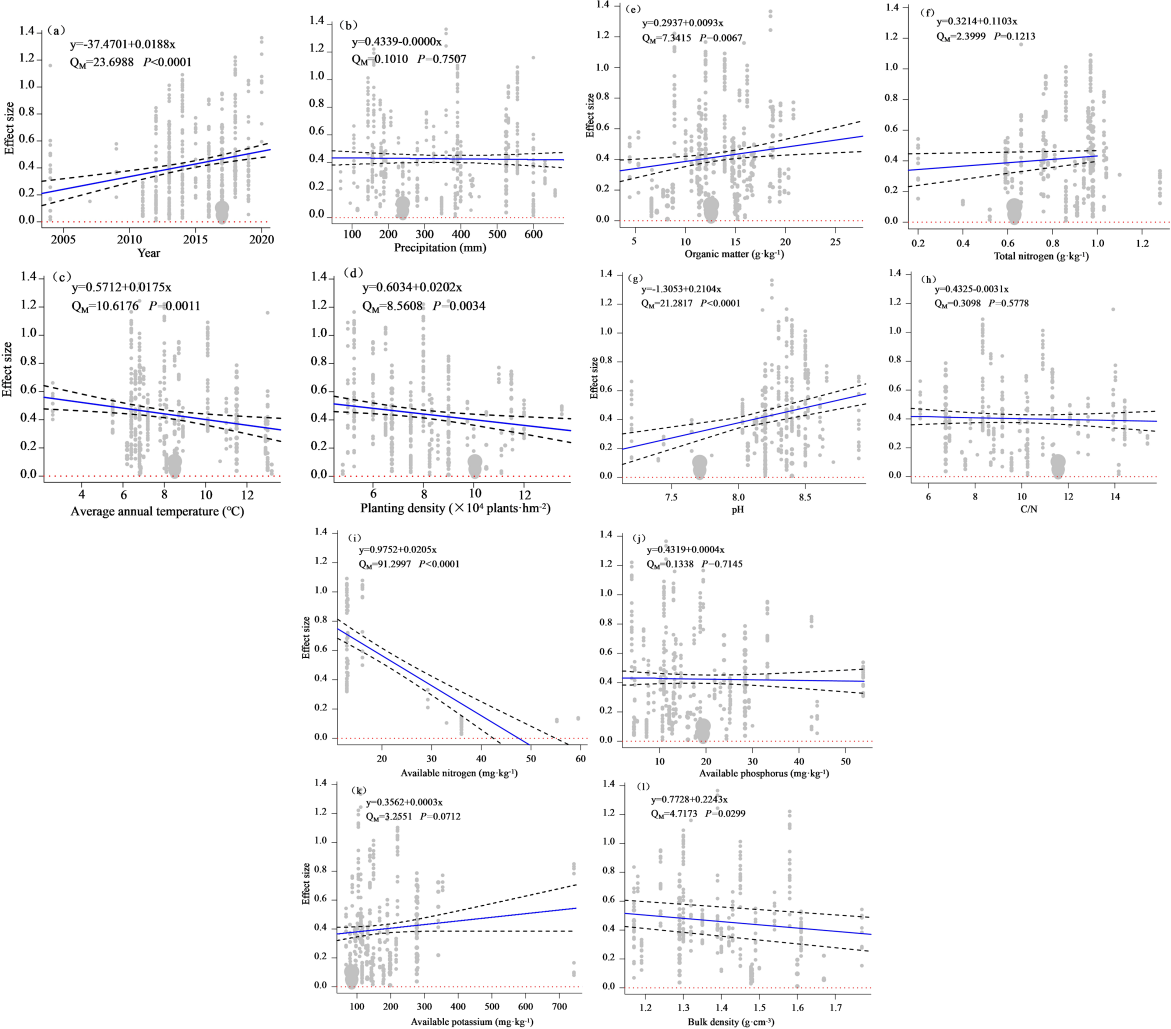
Meta regression analysis of yield effect size with each influencing factor i.e. year **(A)**, average annual precipitation **(B)**, average annual temperature **(C)**, planting density **(D)**, soil organic matter **(E)**, soil total nitrogen **(F)**, pH **(G)**, the ratio of soil organic carbon content to soil total nitrogen content **(H)**, available nitrogen **(I)**, available phosphorus **(J)**, available potassium **(K)**, and bulk density **(L)** respectively. Q_M_ is the heterogeneity caused by a variable, and *P* represents the level of significance.

To further understand the effects of the experimental year, meteorological factors, planting density, and soil physicochemical properties on maize yield changes upon nitrogen addition, a single-factor meta-regression analysis was performed. As shown in **Figure 9**, the experimental year, AAT, planting density, soil OM, pH, AN, AK, and BD exhibited a significant effect (*P* < 0.05) on maize yield increase upon nitrogen addition. In contrast, AAP, C/N, and AP did not exhibit a significant effect (*P* > 0.05). Additionally, the experimental year, soil OM, pH, AK and TN positively influenced the yield-increasing effects of nitrogen application on maize, suggesting a synergistic effect of soil nutrients on maize yield enhancement through nitrogen application. In contrast, AAT, planting density, AN, and BD exhibited a negative effect on the yield-increasing effects of nitrogen application on maize.

### 3.5 Different factors influencing the importance of maize yield effects

**Figure 10:**
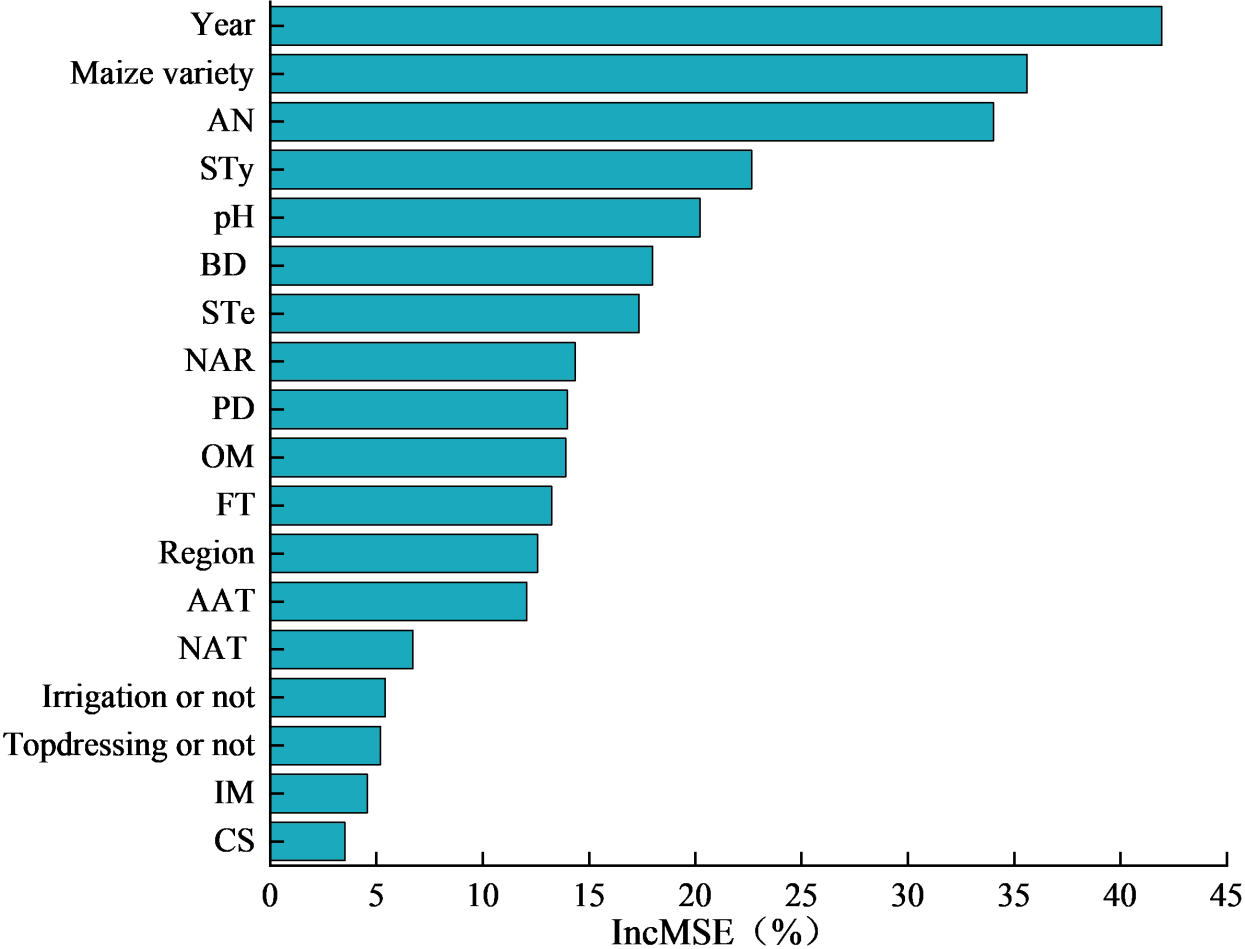
Order of importance of variables. IncMSE is increase in mean squared error, which is determined by randomly assigning a value to each predictor, and if the predictor is more important, the larger the model prediction error is when its value is replaced. Therefore, the larger the value, the higher the importance of the variable. AN stands for Available nitrogen, STy stands for soil type, BD stands for bulk density, STe stands for soil texture, NAR stands for Nitrogen application rate, PD stands for planting density, OM stands for Organic Matter, FT stands for fertilization time, AAT stands for Average annual temperature, NAT stands for Nitrogen application type, IM stands for Irrigation method, and CS stands for Cropping system.

**Figure 10** shows the order of importance of the factors influencing maize yield changes upon nitrogen addition based on the random forest model. The *Q_m_* value and significance of the heterogeneity test of each influencing factor indicated in **Table 2**, **Figure 9** suggested that the soil AK, AP, C/N, TN, and AAP did not significantly affect the changes in maize yield. Hence, they were excluded, and the remaining 18 factors were included in the random forest model to determine the importance of the variables. The model explained 83.7% of the variance, where the experimental year exhibited the highest relative importance (41.95%), followed by maize variety (35.61%), soil AN (34.02%), type (22.64%), pH (20.21%), BD (17.99%), soil texture (17.35%), and nitrogen application (14.34%); the remaining factors exhibited weak relative importance (**Figure 10**).

### 3.6 Identification of the optimal model using multi-factor analysis

**Figure 11:**
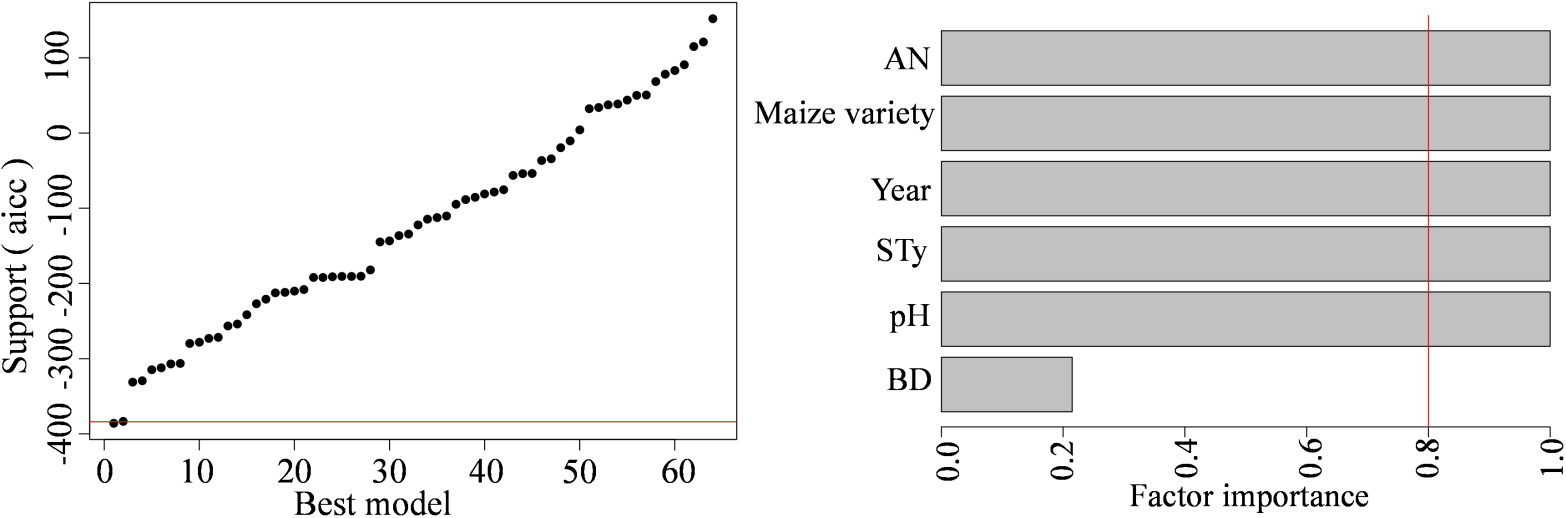
Multi-factor model optimization results and importance of optimal model factors. AN stands for available nitrogen, STy stands for soil type, and BD stands for bulk density.

In this study, multi-factor model optimization was performed for the top 6 factors after random forest model prediction. The model in **Table 3** shows the lowest AICc value and is the optimal model. The weight of this model in the optimal model set was 0.7851, and it considered 85.45% of the heterogeneity sources. A factor importance of > 0.8 suggested that the factor was important (**Figure 11**). Using the model, we noted that soil AN, maize variety, experimental year, soil type, and pH were the important factors affecting maize yield upon nitrogen application.

**Table 3:** Multi-factor optimization model.

Item	parameters
Optimal model	yi~1+Year+Maize variety+STy+AN+pH
Aicc	-385.9291
weights	0.7851
Percentage of heterogeneity considered R^2^	85.45%

AN represents available nitrogen; STy represents soil type.

## 4 Discussion

In this study, we synthesized research cases on the effect of nitrogen addition on maize yield in Northwest China and systematically analyzed the effect of nitrogen addition on maize yield in Northwest China, and concluded that nitrogen addition was able to increase maize yield by 50.26% to 55.72% compared with no nitrogen addition. However, the yield increase effect of nitrogen addition on maize is largely influenced by regional climate, field management and soil environment.

### 4.1 Effects of regional climatic factors on maize yield

Providing adequate nutrients to crops through nitrogen fertilizer application is important to maintain high and stable yields and ensure national food security (Kong et al., 2022). Owing to different climatic characteristics, soil conditions, and field management practices, the increase in maize yield varies greatly among regions. In this study, we found the highest increase in maize yield upon nitrogen application in Gansu province, probably because the crop matured once a year, and plant growth was largely dependent on the local climatic conditions. Gansu province has an arid and semi-arid climate with relatively infertile soils. However, soil fertility was significantly improved upon nitrogen application, and hence, it exhibited the most notable effect on maize yields in the region. Anwar et al. (2009) pointed out that plant growth is highly sensitive to temporal (precipitation and temperature) and spatial (soil) factors. The combination of these factors controls the effectiveness of irrigation and fertilizer application, as well as nitrogen mineralization, during the growing season (Kay et al., 2006). In this study, we found that the highest increase in maize yield upon nitrogen addition was found in the area with an AAP < 200 mm, probably due to the fact that in areas with low precipitation, nitrogen application can alleviate the limitation of maize growth by precipitation to some extent, and at the same time, soil nutrients are relatively scarce in this environment, and maize yields are lowest without nitrogen application, and the most sensitive response to nitrogen addition. In contrast, the increase in maize yield decreased in the area with an AAP of 200–400 mm, and increased in the area with an AAP of ≥ 400 mm. This trend can be explained by the coupling effect of water and nitrogen, and the complex relationship between precipitation and temperature, as suggested by Yamoah et al. (1998), who found that nitrogen fertilizer application was more effective in wet years than in dry years. The regression analysis of precipitation on yield effect size was not significant in this study, which may be attributed to the fact that all the regions were located in Northwest China, which is characterized by relatively low precipitation. Meng et al. (2023) pointed out that when the temperature is 5–10°C, the nitrogen content in the soil is an important limiting factor for maize yield increase, and the increased application of nitrogen fertiliser can significantly promote maize yield. Too high or too low temperature will reduce the enzyme activity in the soil, limit the mineralisation of soil organic nitrogen, and reduce the nitrogen uptake efficiency of maize. Tremblay et al. (2012) found that high temperatures were beneficial in enhancing the response of maize to nitrogen fertilizers, and high average temperatures enhanced microbial activity, which accelerated the rate of nitrogen mineralization. However, high temperatures can also cause excessive nitrogen volatilization losses from the soil, as well as early plant senescence and reduced nutrient accumulation, resulting in reduced yields. Our study indicated that as AAT increased, the positive impact of nitrogen application on maize yield diminished, likely due to the combined effects of high temperatures and low soil moisture, which decreased nitrogen use efficiency. Consequently, this resulted in a decline in maize production. This study also revealed that the increase in maize yield in Northwest China exhibited a fluctuating, rising trend with year progression. This can be explained by the fact that the application of chemical fertilizers has been maintained at a high rate since the latest reforms in China. Some studies have shown that in 2018, the application of chemical fertilizers in China was still as high as 340.77 kg·ha^-1^, and the application of nitrogen fertilizer reached 124.50 kg·ha^-1^ (Zhang et al., 2022c), which improved nutrient availability in the infertile soils of Northwest China, and promoted the improvement of maize yields. Moreover, in recent years, new high-yield maize varieties have been vigorously promoted in China, and the related updates in production technology are inseparable.

### 4.2 Effects of field management on maize yield

Appropriate field management measures create favorable environmental conditions for the growth of maize plants and promote stable and high maize production (Zhang et al., 2022a). Studies have shown that under proper soil moisture conditions, nitrogen recovery and yield are the highest when nitrogen is applied 4–8 weeks after maize planting (Adriaanse and Human, 1993). Zhang et al. (2024) found that maize yield increased with increasing frequency of nitrogen application. In this study, we also concluded that the increase in maize yield was higher with the supplementary fertilizer during the maize-growing period than with the one-time basal fertilizer application, and both of them exhibited similar conclusions. Single-application fertilization during growth stages results in a mismatch between the critical period of crop fertilizer requirement and nitrogen supply, leading to unbalanced nitrogen and nutrient loss during crop growth stages (Xu et al., 2022). Moreover, ample nitrogen supply during the critical growth period after split fertilization can increase the duration of high photosynthetic values and align photosynthetic characteristics with the accumulation of dry matter and nutrients, thereby boosting crop yields (Ma et al., 2007). Overman and Scholtz (1999) found that the nitrogen demand of most crops exhibits an S-shaped curve, and most crops require increased nitrogen application during the middle stage of rapid growth, particularly from the small tassel stage to the tasseling stage in maize. Liu et al. (2023) showed that nitrogen fertilizer application at the JS + anthesis or JS + large tassel + anthesis stage under DI significantly increased maize yield compared to one-time nitrogen fertilizer application at the JS under conventional BI. This finding was consistent with the findings of this study, which suggested that the increase in maize yield upon nitrogen application at the JS + TS or JS + GS, or JS + TS + GS was significantly higher than that at the JS and that the increase in maize yield upon DI was higher than that upon BI. In addition, this study concluded that the highest yield-increasing effect was observed under FI with nitrogen application, probably because under no nitrogen application, maize yield upon FI was significantly lower than that upon other irrigation methods such as DI and BI. In contrast, maize yield under FI was significantly higher after nitrogen application, which resulted in a significant improvement in yield change.

The type and amount of nitrogen fertilizer likewise affects the yield-enhancing effect of maize. Unreasonable nitrogen application is easy to cause nitrogen leaching and volatilization, especially in the maize growing season when precipitation is more concentrated and high temperature (Liu et al., 2022). This study showed that with the increase of nitrogen application, the yield increase effect of maize showed a trend of increasing and then decreasing, and the highest value of the yield increase effect appeared in the nitrogen application of 175–225 kg·ha^-1^. This is consistent with the findings of Li et al. (2021), who found that the yield-enhancing effect of nitrogen application to summer maize increased and then decreased with the increase of nitrogen application, and Wang et al. (2016b), who showed that nitrogen application significantly enhanced the yield of maize, but that there was a decrease in yield when nitrogen fertilizer exceeded 225 kg·ha^-1^. Different types of nitrogen fertilizers have different fertilizer efficacy due to their different mechanisms of nutrient release. Ordinary urea, as the most commonly used quick-acting nitrogen fertilizer, can rapidly increase soil nitrate nitrogen content after two weeks of soil application, but it is easy to pollute the environment if it is applied in excess or in an improper way (Ren et al., 2012). Controlled-release urea can better synchronize the nitrogen demand during the growing season, promote crop growth and yield accumulation, and avoid early plant failure (Ye et al., 2013). Organic and inorganic fertilization meets the requirements of sustainable development of modern agriculture, improves soil granular structure, promotes soil microbial vitality and thus increases soil nutrient content, and also reduces the amount of chemical fertilizers (Akiyama et al., 2010). Zhang et al. (2024) concluded that the application of slow-release fertilizer was more beneficial to increase maize yield compared to urea because it was able to maintain the match between nitrogen supply and plant uptake throughout the reproductive period, which differed from the results of this study where the maize yield-increasing effect of urea application was higher than that of controlled-release nitrogen fertilizer, probably because this study was located in northwestern China, where soils are generally infertile, and the fertilizer release after slow-release fertilizer were applied was slower, and could not meet the nutrient demand of the plant quickly, thus the yield-increasing effect was lower. This study found that although the maize yield increase effect of applying urea, urea + ammonium nitrate, organic and inorganic fertilization, controlled-release nitrogen fertilizer + urea was higher and there was no significant difference between them, but based on the requirements of sustainable development of green modern agriculture, the nitrogen application methods of organic and inorganic fertilization, controlled-release nitrogen fertilizer + urea are recommended to achieve the increase in maize yield.

In addition, this study concluded that Qiangsheng 51 maize has the highest yield increase, because the planting area is Gansu, the climate is arid, and this maize variety has the advantages of drought resistance, high yield and disease resistance, high seed yield, better adapted to the local environmental conditions, so that the effect of yield increase is significant. The improvement of crop yield is often inseparable from the level of group production, and increasing planting density is an important measure (Zhao et al., 1997). Ma et al. (2008) showed that the optimal planting density for high yield of maize through the coupling of planting density and nitrogen fertilizer was 6.17–6.62×10^4^ plants·ha^-1^, and the appropriate pure nitrogen application was 309.88–569.02 kg·ha^-1^. In this study, it was concluded that when maize was planted with a density of > 5×10^4^ plants·ha^-1^, the maize yield increase effect was significantly enhanced, but thereafter, increasing the planting density, the maize yield increasing effect increased insignificantly. The possible reason for this is that water and fertilizer resources are limited in Northwest China, and at too high planting densities, individual competition for water and nutrients is more intense, producing irreversible damage, increasing the rate of empty stalks, decreasing the number of grains in the ears and the resistance of the crop stalks to topple, which would reduce the rate of yield increase. In this study, the yield increase of maize with nitrogen application in rainfed conditions was higher than that in irrigated conditions, probably due to the generally infertile soil environment in the rainfed areas of the Northwest compared to the irrigated areas, where maize yields without nitrogen application were significantly lower than those in the irrigated areas, and where the application of nitrogen significantly improved the soil nutrient conditions, coupled with the effect of precipitation, which resulted in significantly higher yields of maize. In this study, it was found that the yield-gaining effect of the winter wheat-summer maize rotation system was lower than that of the continuous maize crop. Although the gain of additional winter wheat yield is obtained in the winter wheat-summer maize crop rotation system, this crop rotation system in the case of this study is basically located in high latitude areas such as Shaanxi, which is an arid and semi-arid region with limited total precipitation, and the planting of winter wheat will result in the competition for water, nutrients, and other resources with summer maize, which will result in the reduction of the yield of summer maize. Zhang et al. (2016) concluded that in high-latitude areas, cover crops can be planted in the summer when precipitation and temperature are high, and winter wheat can be planted afterward to solve the contradiction of resource competition.

### 4.3 Effects of soil environment on maize yield

The goal of nitrogen fertilizer management is to ensure an adequate supply of effective mineral nitrogen in the soil throughout the growth period of the crop (Adriaanse and Human, 1993). Thus, crop yield is closely associated with soil nitrogen effectiveness and soil characteristics (water-holding capacity, texture, and fertility) (Armstrong et al., 2009). Kyveryga et al. (2009) found that the response of maize to nitrogen application was slightly affected by soil texture, with inter-annual variability having a greater impact than spatial soil differences. Tremblay et al. (2012) demonstrated that soil texture largely determined soil response to nitrogen, with fine-textured soils (C and SC) responding to nitrogen addition more than medium-textured soils (L and SL). This was consistent with the findings of this study, which indicated that the highest increase in maize yield upon nitrogen application was found in clay soils, which may be attributed to the fact that clay soils are denser than sandy and loamy soils in arid environments, exhibit improved water-locking properties, and are less susceptible to nutrient loss, thereby exhibiting the best yield increase. Different soil textures and types significantly affect nitrogen mineralization capacity and crop yield accumulation, with black soils exhibiting significantly higher nitrogen mineralization than red loam and tidal soils (Wang et al., 2004; Wang and Sun, 2011). Feng et al. (2017) studied continuous spring maize in Jilin Province and found that maize yield in black soil averaged 8623 kg·ha^-1^, significantly higher than that in wind-sand soil. The present study showed that the yield-increasing effects were the most significant in the CS. This was probably because when the experimental area was located in the arid CS-rich area, the higher soil calcium carbonate content reduced the risk of soil acidification by chemical fertilizers, which promoted microbial activation and the increase in soil nutrient content, leading to a significant rise in yield change (Gao et al., 2023). Wang et al. (2004) revealed that the higher the soil OM content, the greater the amount of soil nitrogen mineralization. The present study also noted that the yield-increasing effects of nitrogen application gradually increased with the increase in soil OM content.

Changes in soil bulk density can lead to changes in water, fertilizer, air and heat in the soil, which in turn affects the formation of crop yields (Chen et al., 2019; Minhas et al., 2023), and it was found that the range of soil bulk density suitable for crop growth is 1.2–1.3 g·cm^-3^ (Suuster et al., 2011). This is similar to the present study which found that the highest soil bulk density for yield increasing effect of nitrogen added maize was 1.2–1.4 g·cm^-3^. In addition, in our study, when the soil bulk density exceeded 1.4 g·cm^-3^, the yield-increasing effects of N application on maize began to decline significantly, which is consistent with Cui et al. (2024) finding that increasing soil bulk density had a negative effect on maize yield, probably because soils with higher bulk densities tended to be more compact, which impeded the growth of the plant’s root system, and at the same time, high bulk density soils were unfavourable to the root system’s uptake of water and nutrients, which led to the crop’s yield reduction. Soil pH is one of the important factors affecting soil nitrification and has a regulatory effect on soil microbial community and soil nutrient transformation. In this study, it was concluded that the yield-enhancing effect of nitrogen application to maize increased gradually with increasing soil pH. Within a certain range, elevated soil pH increases the solubility of soil organic matter, providing a large amount of material rich in carbon and nitrogen groups for microbial activity, thus promoting nitrogen mineralization, and thus increasing maize yield. Use of nitrogen fertilizer in soils with too high a pH can lead to increased volatilization of soil NH_3_, resulting in nitrogen losses affecting crop nutrient uptake and reducing yields (Laegreid, 1999; Zhang et al., 2022a). Soil C/N ratio is an indicator for evaluating soil nitrogen mineralization capacity, low C/N ratio accelerates nitrogen mineralization rate and soil microbial decomposition, while high carbon to nitrogen ratio produces inhibitory effects (Ge et al., 2013). In this study, we found that the yield increasing effect of N application to maize in Northwest China was higher at low carbon to nitrogen ratio. If the soil C/N ratio increases, it will lead to increased competition for nitrogen between the crop and the microorganisms in the soil (Gunther and Holger, 2003), and this increased competition will limit the amount of nitrogen released from the soil, which will further lead to a decrease in the nitrogen content of the crop leaves, a decrease in the rate of photosynthesis, and a decrease in yield (Gao et al., 2021). In addition, this study found that the highest yield increasing effect of N application to maize was found at the time when the soil quick-acting N content was low. At the time of low soil fertility, the soil demand for nitrogen fertilizer is high, when the appropriate amount of nitrogen fertilizer supply will instead make the yield increase effect higher. Phosphorus and potassium elements are large amounts of essential elements for crop growth and development, and play an important role in plant yield and quality formation (Qian et al., 2023). Shi et al. (2020) concluded that low soil potassium content would reduce the plant photosynthetic rate, thus affecting yield accumulation. Chen et al. (2020) found that appropriate phosphorus application would significantly enhance seed yield of wheat. This is in agreement with the present study which found that the yield-increasing effects of N application in maize were higher in areas with higher soil available phosphorus and available potassium. In our study, soil organic matter, pH, available potassium and total nitrogen all showed positive effects on the yield-increasing effects of N application on maize, reflecting a complex interaction effect between soil nutrients on maize growth, which is similar to Zhang et al. (2024) conclusion that high nutrient soil conditions (organic matter >15 g·kg^-1^; total nitrogen >1.5 g·kg^-1^) are the least restrictive to crop growth and development.

## 5 Conclusions

In this study, a meta-analysis was used to quantitatively investigate the effects of nitrogen addition on maize yield and its primary determinants in Northwest China. We found that nitrogen addition significantly increased maize yield by 53.04% in Northwest China, and the appropriate range of nitrogen application was 175–225 kg·ha^-1^. The analyzed data was highly heterogeneous (*Q_t_* = 44580.2262, *P_Q_* < 0.0001). After introducing explanatory variables using the random forest and multivariate optimality-seeking models, the effect of nitrogen addition on maize yield was mainly attributed to experimental year, maize variety, soil type, AN, and pH. Thus, we concluded that nitrogen must be added to maize cultivated in Northwest China and similar ecological zones to achieve sustainable, high yields while considering climatic conditions, field management practices, and soil environmental factors. Future research could investigate optimal nitrogen application strategies for various environmental conditions and evaluate how precision agriculture techniques enhance nitrogen fertilizer efficiency while minimizing environmental harm. Research should also focus on the effects of climate change on maize production potential in Northwest China and the formulation and assessment of adaptive management practices.

## Data Availability

The original contributions presented in the study are included in the article/supplementary material. Further inquiries can be directed to the corresponding authors.
